# Can we measure iron overload in the heart using *in vivo* MRI T2*?

**DOI:** 10.1186/1532-429X-11-S1-P133

**Published:** 2009-01-28

**Authors:** Taigang He, John-Paul Carpenter, Sutipong Jongjirasiri, Jiraporn Laothamatas, Suthat Fucharoen, David Firmin, Dudley Pennell

**Affiliations:** 1 CMR Unit, Royal Brompton Hospital and Imperial College London, London, UK; 2 Ramathibodi Hospital, Bangkok, Thailand; 3 Thalassemia Research Centre, Mahidol University, Bangkok, Thailand

**Keywords:** Iron Overload, Thalassemia, Thalassemia Patient, Liver Iron, Myocardial Biopsy

## Introduction

The measurement of cardiac iron is important for preventing heart failure and monitoring iron-chelating treatment in thalassemia patients. An MRI T2* technique has been developed and clinically validated for this purpose [1–3]. A recent advance of a black blood T2* sequence shows improved reproducibility [4] and accuracy [3]. However, this technique has not been calibrated against myocardial biopsy. Apart from ethical problems, biopsy is not ideal for assessment of myocardial iron overload because small samples of heterogeneous iron deposition may not accurately reflect the segmental iron content in the myocardium. An alternative approach would be an autopsy study in hearts donated after death or after cardiac transplantation in thalassemia patients. This autopsy study is ongoing aiming at calibration of MRI T2* against iron concentration in the heart [5]. There remains a particular issue as to, however, whether the in vivo T2* measurements equal ex vivo measurements.

A recent study [6] has suggested a significant difference between the in vivo and ex vivo measurement of R2* (1/T2*). The reasons for this discrepancy may not be clear, however, there existed body/tissue temperature difference in the reported study, which can contribute 1.5% per degree Celsius to this discrepancy [7]. In addition, the model used for T2* measurement may give rise to further errors [3].

## Purpose

To compare in vivo and ex vivo myocardial T2* measurements at a comparable temperature and using the correct model.

## Methods

A 23-year-old Thai female thalassemia majorpatient with longstanding cardiac and liver iron overload was studied. The patient underwent MRI scan using the bright blood T2* sequence [2] (twice for reproducibility) in Bangkok on a 1.5 T clinical scanner (GE Signa), again in London 9 days later on a 1.5 T one (Siemens Sonata), using the same sequence. The patient was also scanned using the black blood T2* sequence [4]. The patient died two weeks later. With full ethical approval, the donated heart was formalin fixed before being sliced axially. A custom-made Perspex plinth was used to hold each of the slices which were then scanned at 37°C. Main perameters [2] are TEs from 2.47 ms–16.9 ms (increment 2.01 ms) and a resolution of 1.2 × 1.2 × 5 mm^3^.

## Results

**Figure 1:**
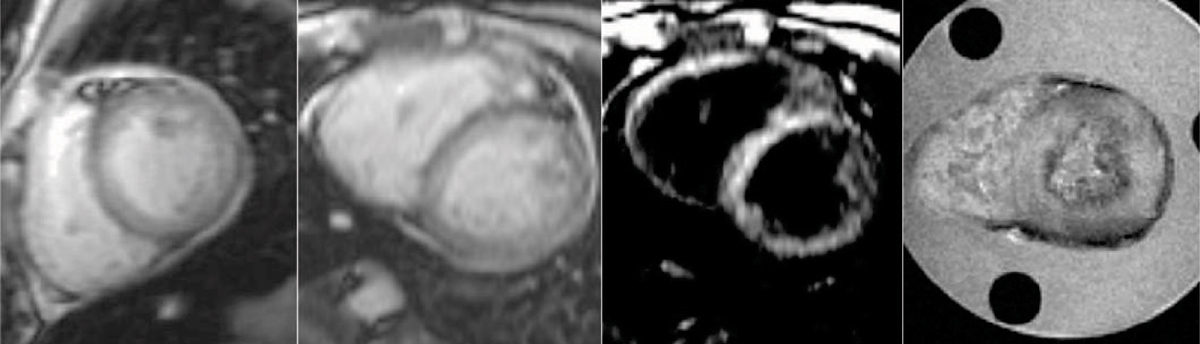
**Example images (from left to right) acquired in Bangkok (TE = 2.1 ms), London (bright and black preparation, TEs = 2.2 ms), and** ***ex vivo*** **scan of a mid-ventricular slice (TE = 2.5 ms), respectively**.

**Figure 2:**
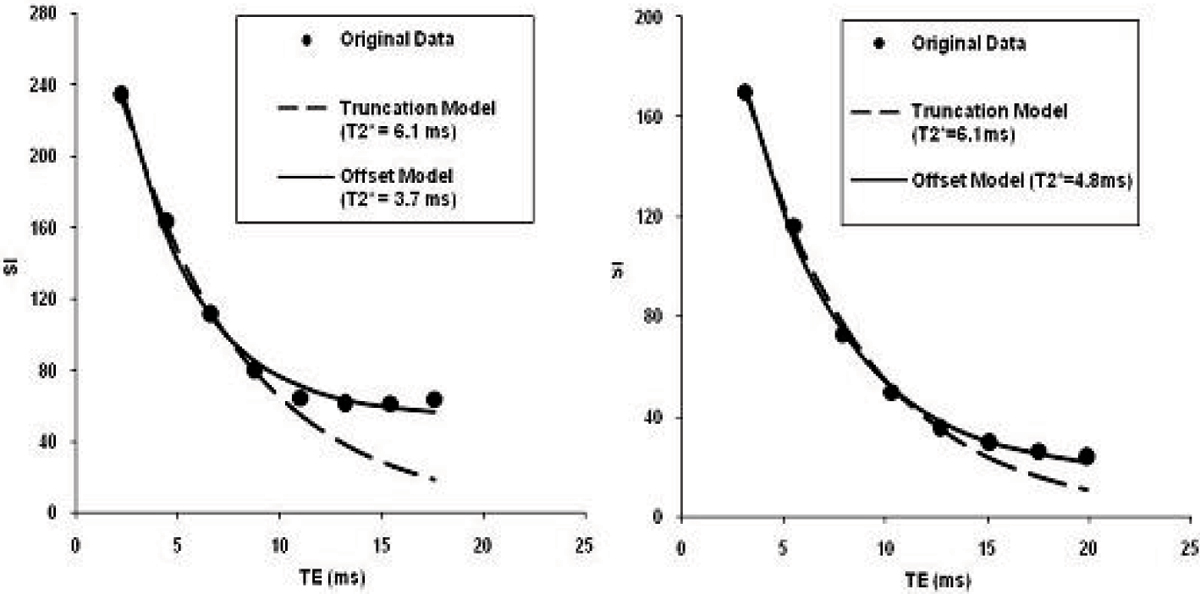
Examples of T2* curve fitting using both truncation and offset models. *Left*: In vivo scan data; *Right*: Ex vivo heart data.

Figure 1 shows example images acquired at Bangkok and London including the ex vivo scan. Figure 2 shows two curve fitting examples demonstrating the discrepancy in T2* measurement using both the truncation and the offset model. The in vivo and ex vivo measurements, however, agree well using the truncation model. Table 1 summaries all T2* measurements using different models. The mean value and standard deviation are also calculated.

**Table 1:** Summary of T2* measurements using both truncation and offset models

T2* (ms)	Bangkok Baseline	Bangkok Repeat	London Bright Blood	London Black Blood	ex vivo	Mean	STD
Truncation Model	6.0	6.1	6.1	6.0	6.1	6.1	0.1
Offset Model	4.6	6.1	3.7	4.6	4.8	4.8	0.9

## Conclusion

This study has demonstrated the good reproducibility of the myocardial T2* measurement using the truncation model, where an in vivo T2* measurement from the patient data agrees with ex vivo one of the fixed heart. These data, along with the calibration study [5], support the clinical use of myocardial T2* in iron overload syndromes.
